# Gender dynamics in diagnostic imaging: Understanding patient preferences and enhancing radiologist preparedness for opposite-gender interactions

**DOI:** 10.3205/000356

**Published:** 2026-05-18

**Authors:** Kanza Riaz, Noshaba Razaq, Muskan Rasheed

**Affiliations:** 1 Wah Medical College Taxila, National University Of Medical Sciences (NUMS), Wah Cantt, Pakistan

**Keywords:** gender dynamics, diagnostic imaging, patient preferences, radiologist preparedness, radiology training, healthcare disparities

## Abstract

**Objective:**

This study explores gender dynamics in diagnostic imaging by identifying factors influencing patients’ refusal of imaging by opposite-gender radiologists and examining radiologists’ preparedness in managing such interactions. The goal is to propose targeted training strategies that enhance gender-sensitive, culturally competent radiology practices.

**Method:**

A cross-sectional survey was conducted among 120 radiology residents in public and private hospitals of Rawalpindi and Islamabad, Pakistan. A structured, paper-based questionnaire was used, comprising two sections: Section A assessed patient-related factors influencing refusal of imaging by opposite-gender radiologists; Section B explored radiologists’ experiences, confidence, and preferences for gender-sensitive training.

**Results:**

Among patients, personal modesty (28.3%), religious beliefs (26.7%), and family influence (24.2%) were the primary reasons for refusing imaging by opposite-gender radiologists. Female patients most frequently cited modesty, while male patients emphasized religion and family. Radiologists reported varied comfort and preparedness in cross-gender interactions. Female radiologists showed significantly greater preparedness (p=.029) but encountered more perceived patient discomfort (p=.008). Preferred training areas included gender-sensitive communication (36.2%) and ethical/legal considerations (31.5%).

**Conclusion:**

Gender-related factors significantly influence patient decisions and radiologist preparedness in diagnostic imaging, especially in culturally conservative settings. Female radiologists appear more prepared but face distinct challenges. Incorporating structured training in gender-sensitive communication, cultural competence, and ethics can improve patient satisfaction and radiologist confidence.

## Introduction

Gender dynamics in healthcare has emerged as an important aspect of patient-centered care, particularly within specialties requiring close physical proximity or sensitive examinations. Physician-patient relationships in healthcare are shaped not only by clinical competence but also by complex social and cultural factors, including physician gender, particularly within culturally conservative societies such as Saudi Arabia and Iran [1], [2]. Gender concordance between patients and physicians significantly influences patient comfort, trust, and satisfaction, especially during intimate medical procedures [3], [4].

A significant proportion of patients prefer physicians of the same gender, particularly in specialties involving physical exposure or sensitive examinations. Alkhaldi et al. [4] reported that 49.5% of patients preferred same-gender surgeons in non-emergency settings, with female respondents significantly more likely to express this preference (p < 0.001) and Sartoretti et al. [5] found that 25% of women undergoing breast ultrasound preferred female radiologists. Important factors influencing these preferences are tied to trust, communication, perceived empathy, emotional safety [6], [7], prior healthcare experiences, patient comfort, particularly during exposure-related procedure [8]. In conservative societies like Saudi Arabia, Iran, and the United Arab Emirates, gender correspondence between patient and provider is particularly significant, influencing clinical outcomes and satisfaction [1], [2], [8]. In Western and multicultural regions, gender and often race continue to impact patient trust and engagement, particularly among historically marginalized populations [7], [9].

There has been extensive research across specialties such as surgery [4], neurosurgery [10] and radiology. Sartoretti et al. [5] demonstrate that while many patients report neutrality in general settings, gender preferences become pronounced during procedures involving physical or emotional vulnerability. Women often prefer female providers in breast imaging and gynecologic contexts, while men may exhibit discomfort with female clinicians in urology and proctology [3]. In diagnostic radiology, the gender of the radiologist can impact perceived comfort, particularly in breast ultrasound and pelvic imaging, though professional behavior and communication often mitigate these concerns [5], [6].

Literature reveals a significant research gap, which indicates gender preferences in areas such as general medicine [3], surgery [4], neurosurgery [10], and nursing care [2] while diagnostic imaging remains underexplored, particularly in terms of gender dynamics, lack of research exploring radiologists’ own preparedness, challenges, and perceptions when conducting cross-gender examinations. Some studies [2], [8] highlight cultural sensitivity, few focus on how these norms specifically affect gender dynamics in diagnostic imaging. Addressing this gap is critical, not only because of the growing emphasis on gender-sensitive medicine [11] but also due to ongoing concerns about gender disparities in radiology workforce composition [12].

This article holds significant importance as it directly addresses the dual objectives of understanding factors influencing patients’ refusal of imaging services from opposite-gender radiologists and identifying targeted training approaches to enhance radiologists’ gender-sensitive care. By focusing on patient comfort and cultural sensitivities specific to radiology, this study fills a critical gap identified in existing literature. Limited attention has been given to radiologists’ own preparedness and professional confidence in managing gender-sensitive interactions. This research helps in training frameworks and policy strategies that promote equitable, culturally appropriate imaging care and improve patient cooperation and satisfaction in cross-gender clinical encounters.

The objectives of the study are as follows:

- To identify the key factors influencing patients’ refusal of imaging services from opposite-gender radiologists
- To identify and recommend targeted training approaches that enhance radiologists’ ability to provide gender-sensitive and professionally confident care in opposite-gender patient interactions

## Research methodology

This study utilized a cross-sectional survey design to gather quantitative data regarding gender dynamics in diagnostic imaging: understanding patient preferences and enhancing radiologist preparedness for opposite-gender interactions. The study population consisted of radiology residents enrolled in an accredited training program in government and private Hospitals of Rawalpindi and Islamabad in Pakistan. A purposive sample of 120 residents was selected to ensure the inclusion of individuals actively engaged in clinical imaging practice and likely to encounter opposite-gender patient interactions. Eligibility criteria included current residency status and voluntary participation. A structured questionnaire for assessing patient preferences and enhancing radiologists’ preparedness was developed to address the study objectives, Section A which is focused on patient perspectives, assessing factors influencing refusal of imaging services from opposite-gender radiologists. Items covered demographics, cultural and religious beliefs, privacy concerns, prior experiences, and trust in professional competence, using a 5-point Likert scale. Section B targeted radiologists, exploring their experiences, confidence, and challenges in opposite-gender interactions. It also assessed current training exposure and preferences for gender-sensitive training approach. The tool will consist of Likert-scale items for quantitative insights.

Data were collected using a structured, self-administered paper-based questionnaire distributed in person to radiology residents across selected teaching hospitals and training institutions. Prior to distribution, the purpose of the study was explained, and informed consent was obtained from each participant. The questionnaires were handed out during academic sessions, departmental meetings, or through coordination with faculty supervisors to ensure accessibility and convenience. Participants were given adequate time to complete the survey on-site or return it within a specified period. 

Ethical consideration was taken from the Institutional Review Board. Data was analyzed using SPSS software, independent T -test samples. Descriptive statistics (frequencies) will summarize participant characteristics. Participation was voluntary, and informed consent was obtained from all respondents. Anonymity and confidentiality were ensured, and no identifiable personal data were collected. 

## Results

Table 1 (Tab. 1) presents the demographic characteristics of the study participants (n=120). The majority of the respondents were late adults (ages 30–40), comprising of 67 individuals (51.5%), while early adults (ages 19–29) accounted for 53 individuals (40.8%). There are 78 (65%) female participants, and 42 males (35%). 90 individuals (69%) are married, followed by 28 singles (21.5%), and 2 participants (1.5%) were separated. In work experience: 35 participants (26.9%) had 1 year of experience, 31 (23.8%) had 2 years, 18 (13.8%) had 3 years, 27 (20.8%) had 4 years, and 8 (6.2%) had 5 years of experience. 

**Figure 1:**
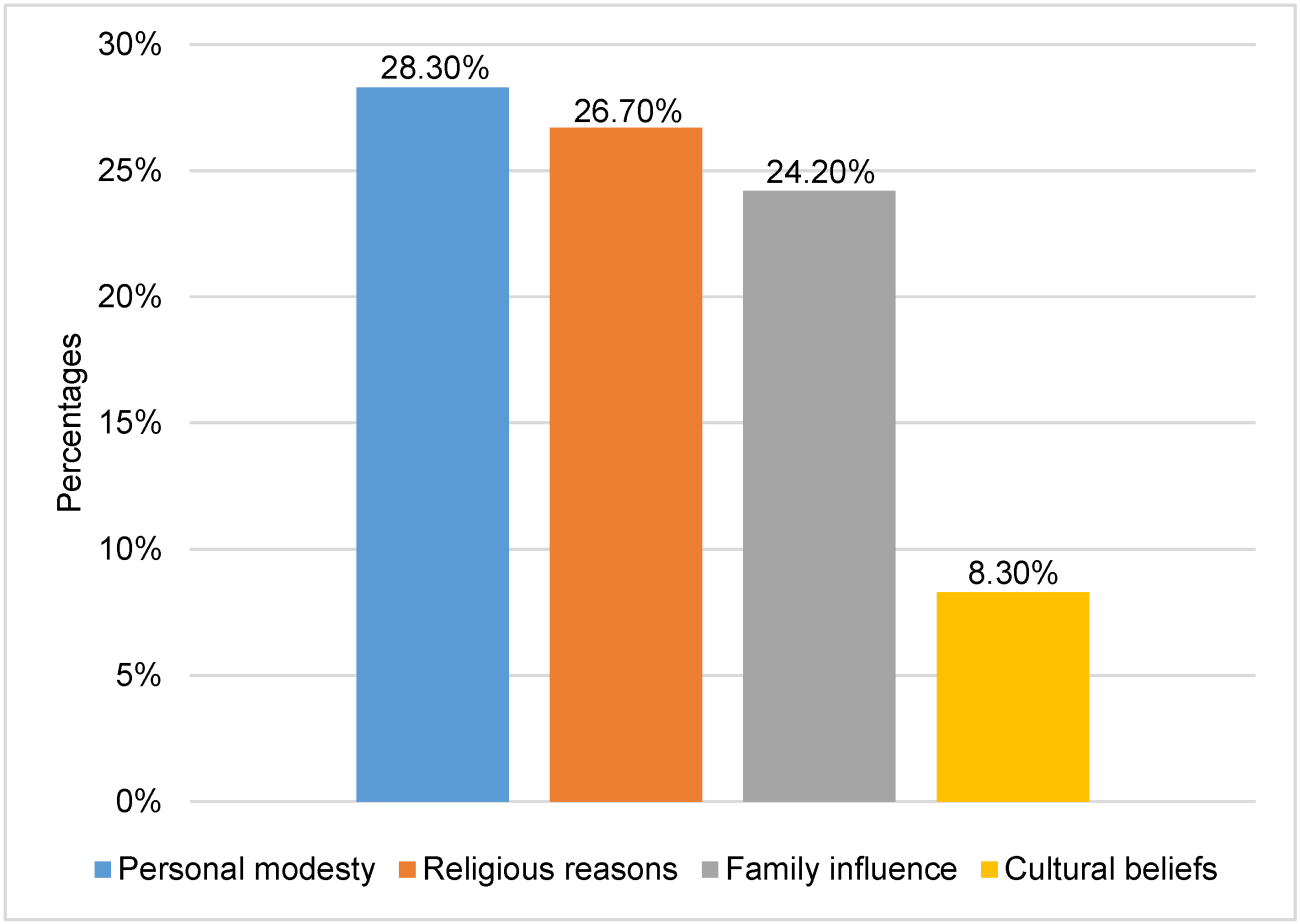
Reasons for patient refusal of imaging by opposite-gender radiologists

Table 2 (Tab. 2) summarizes the patients’ reasons for refusing treatment from the opposite gender. Personal modesty (28.3%) was the most cited reason, followed by religious reasons (26.7%) and family influence (24.2%). Cultural beliefs (8.3%) and unspecified reasons (12.5%) were less frequently reported. To facilitate visual interpretation and comparison of the observed response patterns, the corresponding data are graphically presented in Figure 1 (Fig. 1). 

Table 3 (Tab. 3) shows the relationship between gender and reported reasons. Among female respondents (n=78), the most frequently reported reason was personal modesty (31 cases), whereas male respondents (n=42) most commonly cited religious reasons (16 cases) and family influence (14 cases).

Table 4 (Tab. 4) shows independent samples t-tests was applied in two areas: patient discomfort expressed toward the radiologist t(118)=2.681, p=.008) and radiologist preparedness to handle gender-related cultural issues t(118)=2.212, p=.029). 

Table 5 (Tab. 5) presented training areas that can improve opposite gender interaction, 36.2% preferred training in gender-sensitive communication skills, 31.5% in ethical and legal considerations, 16.9% in cultural competency, 10.8% in simulation-based training, and 4.6% in case studies and best practice discussions. 

## Discussion

This study explored the influence of gender on patient experiences and radiologists’ clinical practices within diagnostic imaging, particularly in a culturally sensitive context. The findings highlight that gender-based discomfort remains a significant barrier to care, with personal modesty, religious values, and familial influence driving patient preferences for same-gender radiologists. These results underscore the importance of considering sociocultural frameworks in imaging services delivery. Radiologists reported experiencing gender-based discomfort and varying degrees of preparedness in addressing gender-sensitive challenges. Female radiologists indicated greater preparedness but also reported heightened awareness of patient discomfort, suggesting a complex interplay between provider awareness and patient perception [13]. Our statistically significant findings including increased patient discomfort with male radiologists (p=.008) and higher preparedness among female radiologists (p=.029) further suggest gendered adaptations in clinical behavior [13]. It has also been shown that female radiologists tend to have a more focused scope of practice but demonstrate greater diagnostic accuracy, underscoring gender-based nuances in clinical work patterns [14]. Increased patient knowledge regarding imaging procedures has been linked to reduced anxiety, highlighting the importance of effective communication in improving patient experience [15]. Furthermore, female physicians are often subject to more critical evaluations by patients, reflecting ongoing gender bias in assessments of technical and interpersonal competence [16].

Statistical analyses from our study revealed significant gender-related trends, including greater patient discomfort with male versus female radiologists (p=.008) and higher preparedness levels reported by female radiologists (p=.029), suggesting adaptive clinical behavior shaped by gender expectations. These observations may be influenced by broader structural barriers that continue to hinder women’s advancement in academic medicine, ultimately shaping their professional training and preparedness [17]. A recurring deterrent for female medical trainees considering radiology is the perception of limited patient interaction, reinforcing the need for proactive mentorship and early exposure to the field to foster gender diversity [18].

Male providers performing intimate imaging procedures are sometimes perceived as infringing upon cultural or personal boundaries, a perception that reinforces the importance of gender sensitivity in clinical care. Related findings indicate that flexible and remote work options have a disproportionately positive impact on female radiologists, potentially supporting better work-life integration and mitigating gender role related constraints [19]. The study’s strengths include its comprehensive quantitative methodology, cross-tabulated gender analyses, and dual focus on patient and provider perspectives, which together enrich the understanding of gender-related challenges in diagnostic imaging. Based on previous studies which emphasize that formalized training in imaging-based screening, including cultural competence, enhances radiologists’ public health leadership, it further supports the need for gender-sensitive educational interventions, suggesting that when radiologists are properly trained in these areas, they can play a more active and informed role in public health. This means they can help improve patient outcomes by ensuring that screening is done effectively, ethically, and based on the latest medical knowledge [20].

## Conclusion

This study demonstrates that personal modesty, religious beliefs, and family influence are key drivers behind patients’ refusal of imaging by opposite-gender radiologists, with female patients predominantly citing modesty and male patients emphasizing religion and family factors. Female radiologists reported greater preparedness but also encountered higher perceived patient discomfort, highlighting a complex dynamic in gender-sensitive clinical interactions. Significant differences in patient discomfort and provider preparedness suggest that gender shapes both patient attitudes and radiologist responses within this cultural context. These findings provide the urgent need for targeted gender-sensitive training in communication and ethics, alongside policy adjustments to accommodate patient preferences without compromising access, thereby improving both patient satisfaction and provider confidence in diagnostic imaging services.

### Limitations and recommendations for future research

The sample was limited to a single geographic region in Southeast Asia (Pakistan), potentially restricting generalizability. Self-reported data may be susceptible to social desirability bias, particularly regarding sensitive topics like modesty and religious beliefs. The study did not incorporate qualitative interviews, which could have enriched understanding of nuanced attitudes and beliefs. Future studies should adopt a longitudinal and multicentric design to capture broader demographic variations. Qualitative exploration through patient and provider interviews would add depth to understanding underlying beliefs and coping strategies. It would also be beneficial to assess the impact of specific interventions, such as communication training or cultural competency modules, on patient satisfaction and provider efficacy.

## Notes

### Competing interests

The authors declare that they have no competing interests.

## Figures and Tables

**Table 1:**
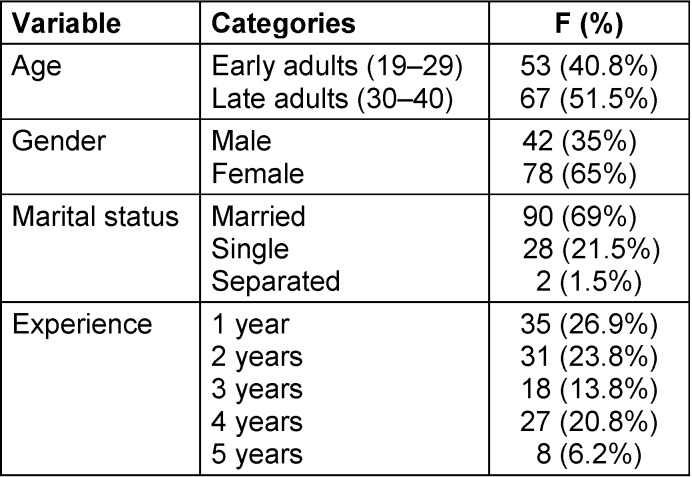
Demographic characteristics of the study participants (n=120)

**Table 2:**
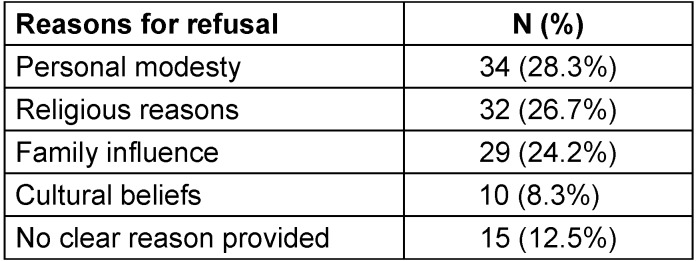
Distribution of reasons provided by patients for refusal of treatment from opposite gender

**Table 3:**
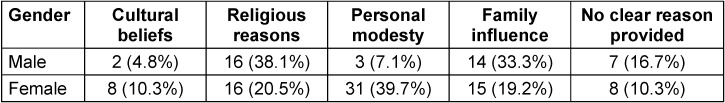
Cross-tabulation of patient gender and reasons for refusal of imaging by opposite-gender radiologists (n=120)

**Table 4:**
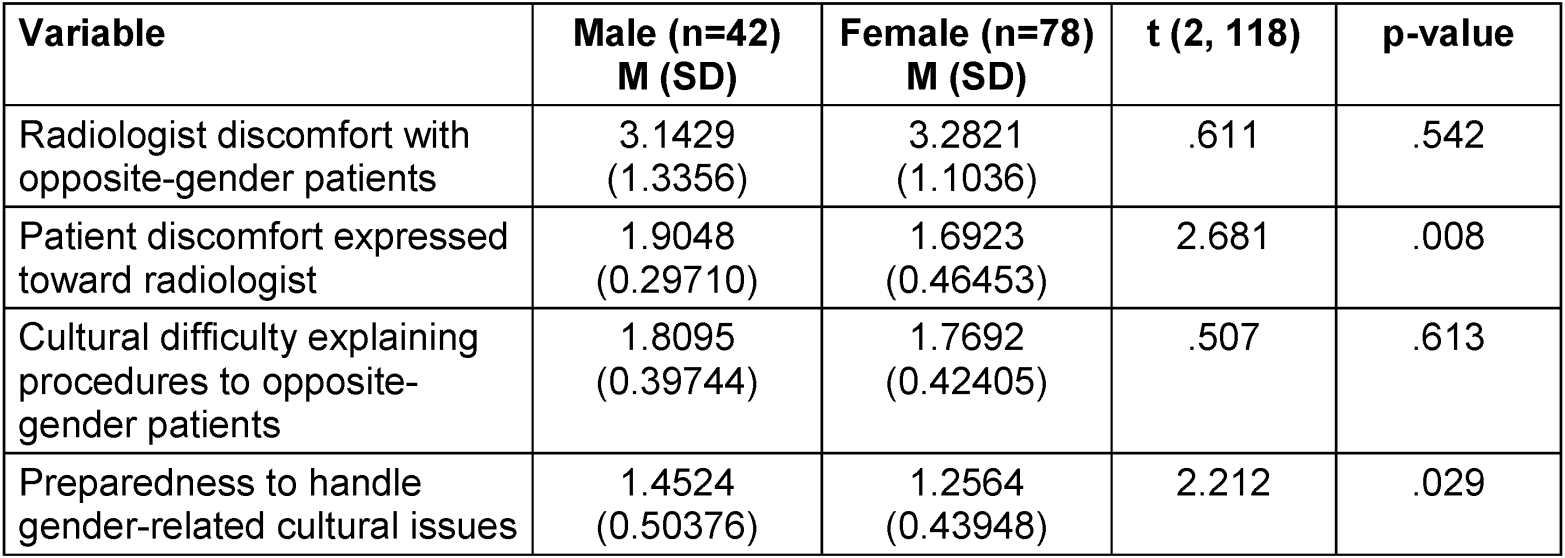
Gender-based differences in radiologist experiences

**Table 5:**
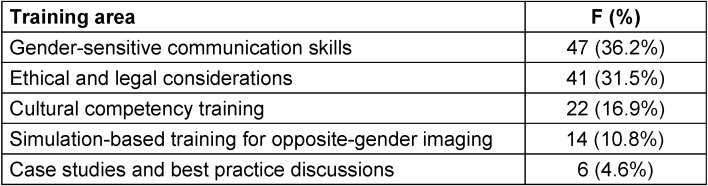
Preferred training areas to improve handling of opposite-gender patients (n=120)
